# Distribution of organic and inorganic mercury across the pelts of Canadian river otter (*Lontra canadensis)*

**DOI:** 10.1038/s41598-019-39893-w

**Published:** 2019-03-01

**Authors:** Kristin M. Eccles, Eric S. Littlewood, Philippe J. Thomas, Hing Man Chan

**Affiliations:** 1 Department of Biology, University of Ottawa, 180, Gendron Hall, 30 Marie Curie, Ottawa, ON K1N 6N5 Canada; 2 Science and Technology Branch, Environment and Climate Change Canada, National Wildlife Research Center, 1125 Colonel By Drive, Raven Road, Ottawa, ON K1A 0H3 Canada

## Abstract

Fur is a common biomarker of environmental mercury (Hg) exposure. Further, there are well-established relationships between total mercury (THg) in fur and organs. However, these models assumed that THg is uniformly distributed across the fur in a pelt. In this study, we assess the distribution of THg and methylmercury (MeHg) across the pelts of four river otters *(Lontra canadensis)*. THg concentrations were measured in the topcoat (n = 95) and undercoat fur (n = 95). MeHg was measured in a subset of these samples (n = 10). Patterns of THg and MeHg were explored using cluster analyses and ANOVAs. Significant differences existed between THg in topcoat and undercoat and between anatomical region (head/body/tail/legs) and fur regions (dorsal/ventral/furline). The cluster analysis showed significant THg clusters in undercoat fur and to a lesser extent topcoat fur. Further, the error rate for predicting internal THg is lowest in the forebody region of the topcoat, thus, making this the optimal region to sample for biomonitoring. Fur samples taken outside of this region could result in prediction error as high as 140% when estimating internal organ THg. The ratio of MeHg in THg in topcoat fur was measured at 95.7 ± 3.4% indicating THg concentrations can be used to assess MeHg exposure.

## Introduction

Mercury (Hg) is an environmental pollutant of global concern found in terrestrial and aquatic ecosystems. The methylated form of Hg (MeHg) is bioavailable and readily accumulates in the kidneys, liver, central nervous system and produce adverse effects on immune response, structure and function of nervous tissues, fertility, and fetal development in humans and wildlife^1–3^. In North American freshwater ecosystems, river otters (*Lontra canadensis*) are good sentinel species for MeHg exposure because of their non-migratory, non-hibernating behavior, their small home range, as well as their high year-round fish consumption^4,5^. These top predators are reliable indicators of environmental contamination as tissue Hg concentrations are correlated with Hg levels in fish prey species from the same watersheds^6^.

Tissues examined in previous biomarker studies of Hg exposure in river otters include brain, liver, kidney, muscle and fur. Mercury concentrations in all tissues are directly related to the concentrations of mercury found in the blood and fur^7–9^. Hair is advantageous because it is a minimally-invasive matrix and an important excretory pathway which provides temporal trends in Hg exposure at concentrations that can be upwards of two orders of magnitude higher than those in blood^10^. Since both MeHg and Hg^2+^ ions have high affinities for thiol groups, the high sulfur content and slow growth rate of fur make this keratinous matrix ideal for assessing temporal trends of Hg exposure between seasonal molts of topcoat and undercoat fur^11^. Previous studies have also reported that total Hg (THg) concentrations in otter fur are strongly correlated with those concentrations found in the brain^8^, liver^8,12^, kidney^13^, and muscle tissue^13^.

Using fur to estimate THg exposure in river otters is similar to the well-established methods for using hair to estimate human THg exposure^4,14^. Several studies have discussed the utility of fur THg concentrations to estimate the internal organ THg concentrations in these river otter and mink^15,16^, using predictive regression models. The accuracy of estimates generated by this model vary between 28.6–45.4% based on a normalized root mean squared error (NRMSE) for river otter depending on the tissue^9^. While these relationships did not appear to be age or sex dependent^8,16^, some of the unexplained variance could be attributed to differences in fur sample locations within each animal. A survey of the literature identified eight studies that reported fur THg sampled from a variety of locations (commonly on the limbs) (Table 1).

**Table 1:** Summary of fur sample locations in Hg biomonitoring studies in river otter.

Study	Sample Location
Halbrook *et al*.^41^	Unspecified Sample Location
Evans *et al*.^12^	Unspecified paw
Evans *et al*.^28^	Unspecified paw
Fortin *et al*.^15^	Forelimbs
Yates *et al*.^16^	Unspecified Sample Location
Strom^18^	Hind paw
Klenavic *et al*.^8^	Between the footpads
Dornbos *et al*.^42^	Unspecified paw

Few studies have assessed the potential difference in THg concentrations in different regions of the pelt. Klenavic *et al*.^8^, assessed the differences between the fur THg concentrations from all four footpads and concluded that no significant differences existed. Wilkie *et al*.^17^ assessed fur THg concentrations on the dorsal surface of all paws, the dorsal base of the skull, the middle dorsal surface, the middle ventral surface, and the base of the tail, in river otters from central Saskatchewan, Canada. This study concluded that there were significant differences between sampling locations but there were no difference in THg concentrations between the four paws. However, this study did not distinguish between topcoat or undercoat fur^17^.

Biomonitoring programs using human hair have an established standard protocol of sampling from the occipital region^18^. In contrast, there is no standardized fur sampling location for biomonitoring in furbearing mammals^19–23^. Given the inconsistencies in sampling procedures for fur bearing mammals, and the potential for variation of THg concentrations across a pelt, we hypothesize that some of the unexplained variance in the regression models relating fur THg concentrations to the internal organ THg could be the result of a heterogeneous distribution of THg across the pelt. Further differences could exist between the concentrations of THg observed in the topcoat and undercoat layers in the analyzed fur samples. If significant differences in accumulation patterns of THg exist across different regions or hair types of a pelt, then the generated predictive models for Hg concentrations could result in biased estimates.

The objective of this research is to characterize the variability of excreted Hg compounds in otter pelts in order to identify the fur type (topcoat/undercoat) with the least variability and to determine if excretion of Hg compounds is dependent on anatomical region. Further, we will examine the relationship between the concentration of THg and internal organ THg to suggest an optimal sampling location as a standardized sampling procedure for future biomonitoring programs.

## Results

### THg in Individual Pelts

**Figure 1:**
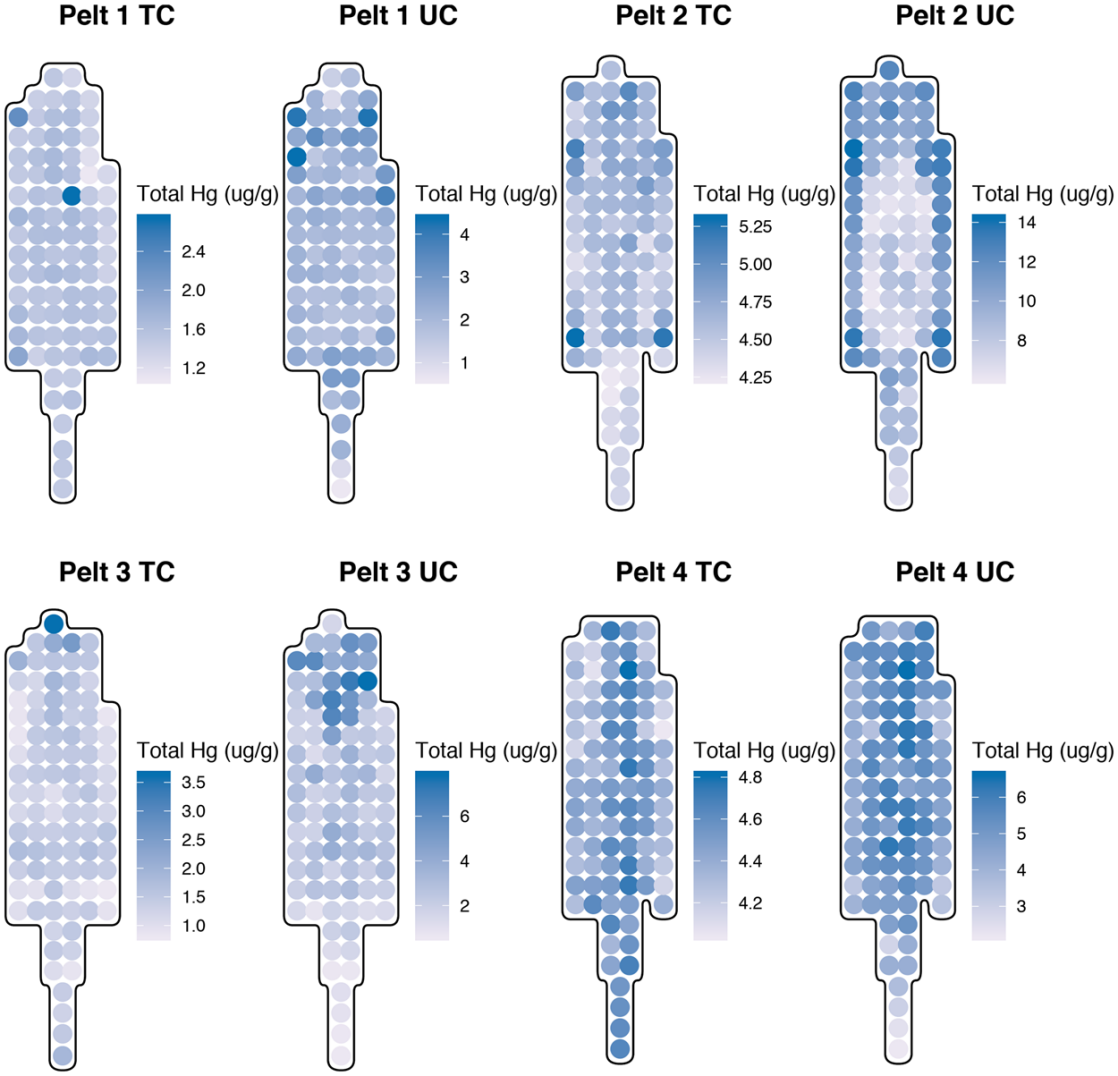
Distribution of THg concentrations in topcoat (TC) and undercoat (UC) fur in individual pelts.

The measured THg and MeHg data for each pelt is summarized in Table 2 and a visual representation of the THg concentrations across the topcoat and the undercoat are summarized in Fig. 1. These plots illustrate the variability of THg in topcoat and undercoat fur. Quantitatively, a Welch’s paired t-test showed that the concentrations of THg in topcoat fur were significantly lower than THg concentrations in undercoat fur sampled from the same location in all pelts (p < 0.001). Pearson product-moment correlation coefficients indicated that a weak positive correlation (*r* ≤ 0.35, p < 0.01) existed between topcoat and undercoat fur THg concentrations in individual pelts and that these were statistically significant for pelt 2, pelt 3, and pelt 4.

**Table 2:** Summary of total mercury (THg) in the topcoat and undercoat, and methylmercury (MeHg) for each otter pelt, measured in μg/g.

Pelt	Mercury species	n	Mean	SD	Min	Max
Pelt 1	THg Topcoat	89	1.55	0.20	1.08	2.74
	THg Undercoat	89	2.19	0.62	0.61	4.37
	MeHg Topcoat	10	1.58	0.72	0.82	3.41
Pelt 2	THg Topcoat	98	4.60	0.20	4.23	5.30
	THg Undercoat	98	9.42	2.20	6.01	14.21
	MeHg Topcoat	10	4.54	0.59	4.08	6.11
Pelt 3	THg Topcoat	96	1.42	0.37	0.81	3.63
	THg Undercoat	96	2.86	1.51	0.62	7.84
	MeHg Topcoat	11	1.44	0.75	0.41	3.30
Pelt 4	THg Topcoat	95	4.44	0.16	4.04	4.81
	THg Undercoat	95	4.87	0.90	2.18	6.61
	MeHg Topcoat	9	4.44	0.16	4.12	4.59

Min = variable minimum, Max = variable maximum, SD= standard deviation.

**Figure 2:**
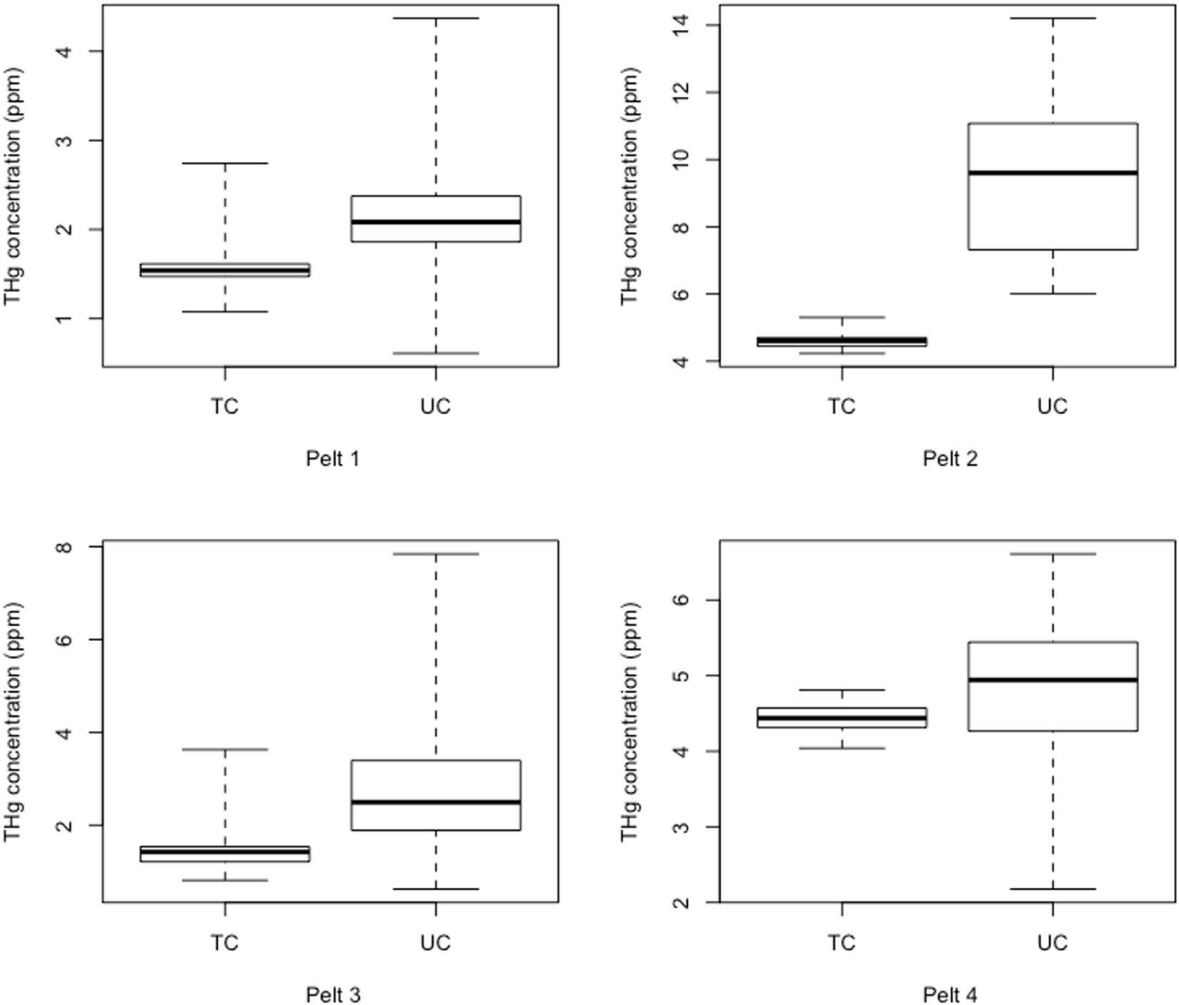
Boxplots of total mercury (THg) concentration ranges in topcoat (TC) and undercoat (UC) fur for individual pelts. In these boxplots, the thick black line is the median, the boxes represent the interquartile range and the whiskers span the range of THg concentrations.

Relative to the topcoat, the range of THg concentrations in the undercoat is 2.26, 7.66, 2.56, and 5.75 times higher in each of the pelts respectively (Fig. 2, Supplementary Table S1). Further, the variance of undercoat fur THg concentrations were 10–118 times higher than the topcoat THg fur variances (Supplementary Table S2).

There is a statistically significant difference in fur THg concentrations between the anatomical regions where fur is sampled (i.e. from the head, body, legs or tail) in some pelts. Significant effects of the anatomical region in topcoat fur THg concentrations existed in pelt 2 (F_3,94_ = 9.831, p < 0.001) and pelt 3 (F_3,92_ = 14.31, p < 0.001) and was significant in the undercoat of pelt 2 (F_3,94_ = 3.088, p = 0.031), pelt 3 (F_3,92_ = 9.453, p < 0.001) and pelt 4 (F_3,91_ = 8.482, p < 0.001). A post-hoc Tukey comparison of THg concentration between anatomical regions in the topcoat and undercoat fur is presented in Table 3. The magnitude of statistically significant differences ranged from 0.3–1.1 μg/g in topcoat fur and 1.2–2.6 μg/g in undercoat fur. The location of the anatomical regions on each pelt can be found in Supplementary Fig. S7.

**Table 3:** THg concentration differences between the levels of the anatomical region factor for topcoat and undercoat fur.

Pelt		Topcoat Difference (μg/g)	Adj. p-value	Undercoat Difference (μg/g)	Adj. p-value
Pelt 1	Head-Body	−0.1 ± 0.2	0.412	−0.5 ± 0.7	0.305
	Leg-Body	0.1 ± 0.3	0.724	0.1 ± 0.8	0.99
	Tail-Body	−0.1 ± 0.2	0.815	−0.1 ± 0.5	0.993
	Leg-Head	0.2 ± 0.3	0.248	0.6 ± 1.1	0.477
	Tail-Head	0.1 ± 0.3	0.869	0.4 ± 0.9	0.557
	Tail-Leg	−0.2 ± 0.3	0.496	−0.2 ± 1.0	0.975
Pelt 2	Head-Body	0.1 ± 0.2	0.873	1.9 ± 2.6	0.226
	Leg-Body	−0.1 ± 0.3	0.822	2.6 ± 3.3	0.177
	Tail-Body	**−0**.**3 **±** 0**.**1**	<**0**.**001**	−0.6 ± 1.7	0.758
	Leg-Head	−0.2 ± 0.3	0.642	0.7 ± 4.1	0.972
	Tail-Head	**−0**.**3** ± **0**.**2**	**0**.**002**	−2.5 ± 2.9	0.118
	Tail-Leg	−0.2 ± 0.3	0.348	−3.2 ± 3.6	0.095
Pelt 3	Head-Body	**0**.**9** ± **0**.**4**	**<0**.**001**	0.7 ± 1.6	0.688
	Leg-Body	−0.2 ± 0.4	0.735	**−1**.**9 **±** 1**.**8**	**0**.**036**
	Tail-Body	0.0 ± 0.3	0.996	**−1**.**9 **±** 1**.**1**	**<0**.**001**
	Leg-Head	**−1**.**1** ± **0**.**6**	**<0**.**001**	**−2**.**6 **±** 2**.**4**	**0**.**026**
	Tail-Head	**−0**.**9** ± **0**.**4**	**<0**.**001**	**−2**.**5 **±** 1**.**9**	**0**.**003**
	Tail-Leg	0.1 ± 0.5	0.86	0.0 ± 2.0	1
Pelt 4	Head-Body	0.1 ± 0.2	0.883	−0.1 ± 1.1	0.992
	Leg-Body	0.0 ± 0.2	0.997	−1.0 ± 1.2	0.176
	Tail-Body	0.1 ± 0.1	0.079	**−1**.**2 **±** 0**.**7**	**<0**.**001**
	Leg-Head	0.0 ± 0.3	0.986	−0.9 ± 1.6	0.507
	Tail-Head	0.1 ± 0.2	0.914	−1.1 ± 1.2	0.106
	Tail-Leg	0.1 ± 0.3	0.758	−0.2 ± 1.4	0.977
Composite (normalized THg)	Head-Body	0.001 ± 0.042	1.000	−0.015 ± 0.138	0.991
	Leg-Body	0.007 ± 0.037	0.953	−0.075 ± 0.121	0.370
	Tail-Body	−0.010 ± 0.029	0.801	**−0**.**143 **±** 0**.**094**	**<0**.**001**
	Leg-Head	0.006 ± 0.054	0.991	−0.060 ± 0.176	0.811
	Tail-Head	−0.011 ± 0.049	0.928	−0.127 ± 0.159	0.161
	Tail-Leg			−0.068 ± 0.144	0.612

Difference of the means is presented as the difference ±95% confidence interval. P-values were adjusted for multiple comparison. Composite values have been normalized between zero and one.

Adj. p-value = p-value adjusted for multiple comparisons.

Similarly, we detected a statistically significant difference in fur THg concentrations between fur regions (i.e. from the dorsal, fur line, or ventral regions) in some pelts. Significant effects of fur region on topcoat fur THg concentrations existed in pelt 3 (F_2,93_ = 5.058, p = 0.008) and pelt 4 (F_2,92_ = 53.02, p < 0.001) and significant differences in the undercoat were found in pelt 1 (F_2,86_ = 9.294, p < 0.001), pelt 2 (F_2,95_ = 44.47, p < 0.001) and pelt 4 (F_2,92_ = 4.602, p = 0.012). A post-hoc Tukey comparison of THg concentration differences between each fur region factor in topcoat and undercoat fur is presented in Table 4. The magnitude of statistically significant differences ranged from 0.1–0.3 μg/g in topcoat fur and 0.4–3.3 μg/g in undercoat fur. The location of the fur regions on each pelt can be found in Supplementary Fig. S8.

**Table 4:** THg concentration differences between the levels of the fur region factor for topcoat and undercoat fur.

Pelt	Comparison	Topcoat Difference (μg/g)	Adj. p-value	Undercoat Difference (μg/g)	Adj. p-value
*Pelt 1*	Fur Line-Dorsal	−0.1 ± 0.1	0.135	0.1 ± 0.4	0.624
	Ventral-Dorsal	−0.1 ± 0.1	0.110	**0**.**6** ± **0**.**3**	<**0**.**001**
	Ventral-Fur Line	0.0 ± 0.1	0.989	**0**.**4** ± **0**.**4**	**0**.**017**
*Pelt 2*	Fur Line-Dorsal	0.0 ± 0.1	0.604	−0.1 ± 1.0	0.957
	Ventral-Dorsal	0.1 ± 0.1	0.12	**3**.**2** ± **0**.**9**	<**0**.**001**
	Ventral-Fur Line	0.0 ± 0.1	0.722	**3**.**3 **±** 1**.**1**	<**0**.**001**
*Pelt 3*	Fur Line-Dorsal	−0.1 ± 0.2	0.854	0.1 ± 1.0	0.955
	Ventral-Dorsal	**−0**.**3 **±** 0**.**2**	**0**.**007**	−0.1 ± 0.9	0.986
	Ventral-Fur Line	−0.2 ± 0.2	0.106	−0.2 ± 1.1	0.917
*Pelt 4*	Fur Line-Dorsal	**−0**.**2 **±** 0**.**1**	<**0**.**001**	0.1 ± 0.6	0.959
	Ventral-Dorsal	**−0**.**3 **±** 0**.**1**	<**0**.**001**	**−0**.**5** ± **0**.**5**	**0**.**020**
	Ventral-Fur Line	**−0**.**1 **±** 0**.**1**	**0**.**004**	**−0**.**6** ± **0**.**6**	**0**.**047**
Composite (normalized THg)	Fur Line-Dorsal	−0.012 ± 0.024	0.428	−0.005 ± 0.079	0.989
	Ventral-Dorsal	−0.019 ± 0.020	0.057	**0**.**103** ± **0**.**065**	<**0**.**001**
	Ventral-Fur Line	−0.007 ± 0.025	0.808	**0**.**108** ± **0**.**085**	**0**.**009**

Difference of the means is presented as the difference ±95% confidence interval. P-values were adjusted using the Tukey correction for multiple comparison.

Adj. p-value = p-value adjusted for multiple comparisons.

**Figure 3:**
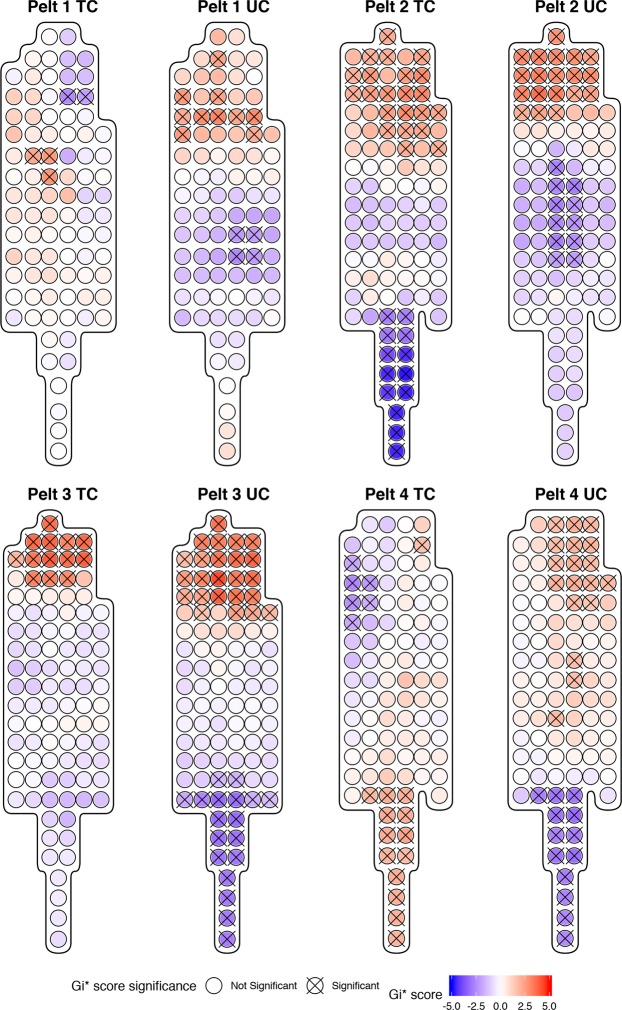
Getis-Ord local Gi* analyses for regions of statistically significant high and low THg concentrations in topcoat (TC) and undercoat (UC) fur of individual pelts. Statistically significant spots at the 95% CI (Gi* = +/−1.96) are identified with an ‘X’.

Fur sampling points on individual pelts with statistically significant clusters of high or low THg concentrations were identified with Getis-Ord local *G*_i_* analyses in Fig. 3. All pelts showed some hotspots (i.e. regions where high values cluster together) and coldspots (i.e. regions of the pelt where low values cluster together). Typically, the undercoat has larger clusters than the topcoat, demonstrating greater heterogeneity of the undercoat in comparison with the topcoat. Most of the pelts demonstrate hotspots around the head region and coldspots around the tail region.

### Methylmercury in Topcoat

The fraction of fur THg present as MeHg was determined by fitting a linear model with a zero intercept. Data where the MeHg in THg ratios exceeded 110% were removed, yielding a final sample size of n = 34. The β-coefficient for this relationship was 0.957 ± 0.034 and relationship was highly significant (R^2^ = 0.99, p-value < 0.001). This linear model met all regression assumptions of residual linearity, homoscedasticity, normality, and no autocorrelation.

### THg in Composite Pelts

**Figure 4:**
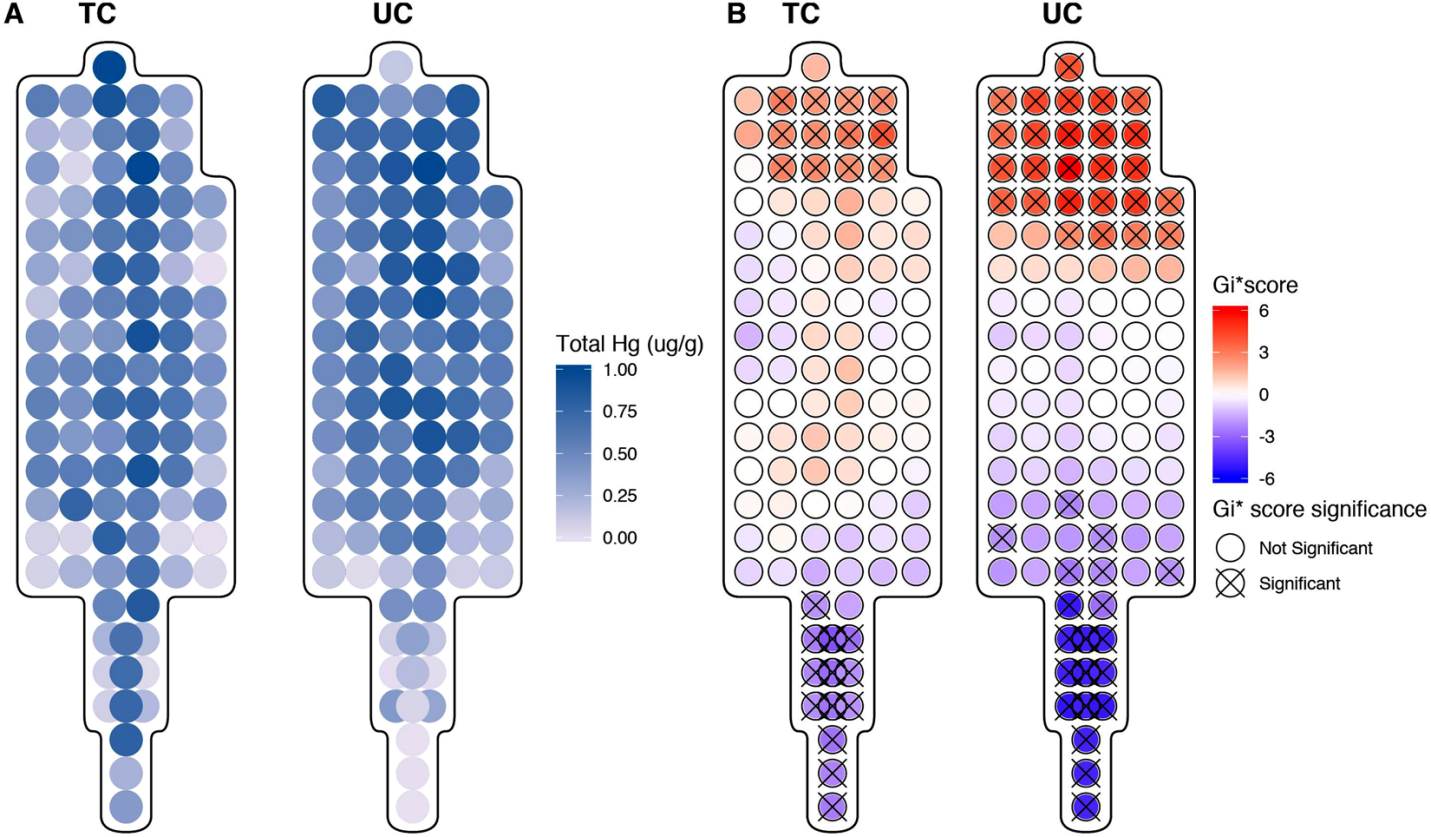
(**A**) Distribution of normalized THg concentrations in topcoat (TC) and undercoat (UC) fur of the generated composite pelt. (**B**) Getis-Ord local Gi* analyses for regions of statistically significant high and low THg concentrations the topcoat and undercoat of the composite pelt. Statistically significant spots at the 95% CI (Gi* = +/−1.96) are identified with an ‘X’.

As the topcoat and undercoat of pelts are going to be subject in interindividual variation, a composite pelt of normalized THg concentrations averaged across the topcoat and undercoat was created. This composite pelt (normalized and averaged pelts) demonstrates average patterns of THg across the topcoat and undercoat (Fig. 4A). Similar to the results of the individual pelts, the mean of the differences of the normalized THg concentrations in the topcoat and undercoat fur was highly significant. A Welch’s paired t-test indicated that average normalized THg concentrations were 0.257 units lower (t_104_ = −19.06, p < 0.001) in topcoat fur compared to undercoat fur. The ratio of the variances of the normalized topcoat and undercoat THg concentrations (ratio = 0.0817, F_104,104_ = 0.0817, p < 0.001) was also significant and indicates that the spread of THg concentrations is larger in the undercoat than in topcoat fur. The range of normalized THg concentrations in undercoat fur (0.061–0.860 units) was 2.88 times larger than the range of topcoat fur (0.063–0.341 units).

The composite pelt dataset showed no significant differences between anatomical regions (F_3,101_ = 0.4167, p = 0.7414) or fur regions (F_2,102_ = 2.837, p = 0.0632) in the normalized topcoat THg concentrations but there was a significant difference in anatomical regions (F_3,101_ = 5.648, p = 0.001) and fur region (F_2,102_ = 8.146, p < 0.001) in the undercoat fur. Tukey post-hoc comparisons of normalized THg concentration differences between anatomical regions and fur regions in the topcoat and undercoat can be found in Tables 3 and 4 respectively.

The results of the Getis and Ord’s Gi* cluster analysis (Fig. 4B) indicate that in the topcoat and bottom coat, there is a statically significant cluster of high values (hotspot) at the head and a statistically significant cluster of cold spots in the tail region (similar to results obtained in individual pelts). The clusters are smaller in the topcoat than in the undercoat demonstrating greater heterogeneity of the undercoat in comparison with the topcoat.

### Optimal Sampling Location

**Figure 5:**
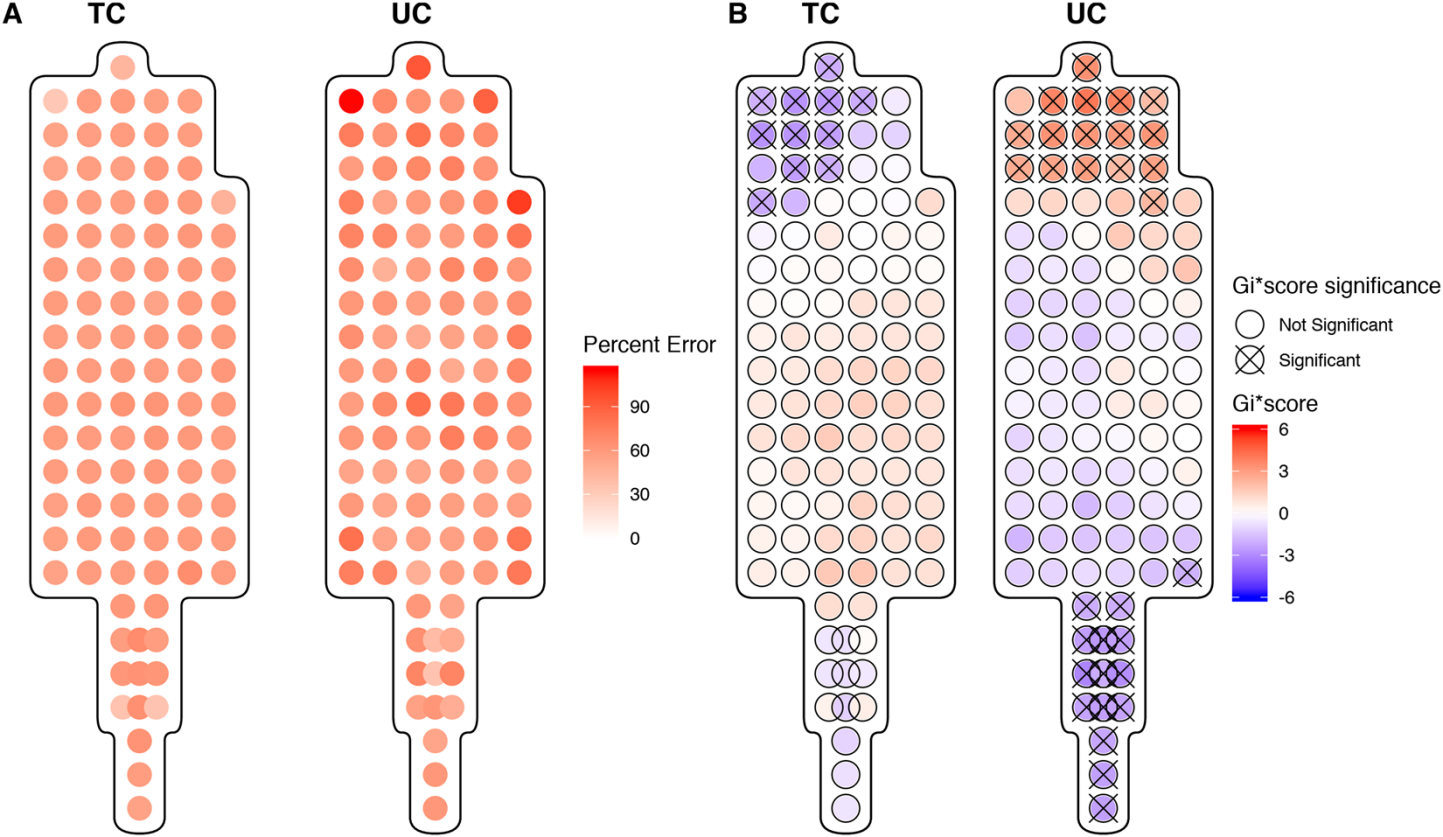
(**A**) Average percent residual error for a weighted combination of residual error from brain, liver kidney, and muscle THg concentration based on fur THg in the top coat (TC) and undercoat (UC). (**B**) Getis-Ord local Gi* analyses for regions of statistically significant high and low residual error in TC and UC. Statistically significant spots at the 95% CI (Gi* = +/−1.96) are identified with an ‘X’.

The percent residual from predicting internal organ THg concentrations from sampled fur THg at each sample point was used to demonstrate the accuracy of the predictor. The distribution of average error across all organs, with brain having a heavier weighting, across all pelts can be seen in Fig. 5 (Left). The average topcoat error ranged from 30% to 89% error and in the undercoat the average percent error ranged from 26% to 143%. The results of the Getis and Ord’s Gi* cluster analysis revealed a cluster of low error rates in the head region of the topcoat (Fig. 5 Right). Conversely, in the undercoat at the head, there is a cluster of high error rates and a cluster of low error rates in the tail. This highlights the variability of error rates in the undercoat and differences between the topcoat and undercoat when using fur THg as a predictor of internal THg concentrations.

## Discussion

Since there is no standardized sampling location for fur in wildlife biomonitoring studies, previous wildlife biomonitoring often collected hair from different regions and assumed that THg is homogenously distributed across the pelt. The results presented in this paper indicate that THg has a heterogeneous distribution across both the topcoat, and to a stronger extent, the undercoat of a pelt in river otter. Further, this is the first study to quantify the difference in THg concentrations in both topcoat and undercoat. The differences between the THg concentrations in paired topcoat and undercoat fur samples were highly significant in all individuals; the concentration of THg in topcoat fur is significantly lower and less variable than the THg in undercoat. Fur sampled from the topcoat consistently had the smallest THg variances whereas undercoat variances were at least 10 times greater in individual pelts. This indicates that the excretion of Hg compounds into undercoat fur is more variable than the excretion into topcoat fur and also suggests that mean topcoat THg concentrations have a greater precision than mean undercoat THg concentrations. As a result, topcoat and undercoat fur should be analyzed separately to avoid error introduced by changes in relative topcoat/undercoat abundance in samples.

Differences in anatomical and fur regions indicated that the variability of THg in topcoat and undercoat in some regions of the individual and composite pelts were statistically different. Overall, in the composite pelt, there was no significant effect of anatomical and fur regions in topcoat fur while differences were detected in undercoat fur. These results suggest that for a pelt with an average THg distribution, the differences between topcoat fur THg concentrations in the head, body, legs or tail may be statistically significant but may not be biologically significant due to small difference in THg between the regions. Individually, undercoat THg concentrations have greater variability than topcoat concentrations and suggest that part of this variability may be explained by anatomical region. The results from this study may explain the significant differences between THg in different anatomical regions in Wilkie *et al*.^17^ since the samples in that study consisted of both topcoat and undercoat fur. It is important to note that paws were discarded during pelt process and therefore not analyzed for THg concentrations in our study. Therefore, it was not possible to assess the THg in the fur in that region which presents itself as a limitation.

There were both interindividual and intraindividual variations of observed values in the distribution of THg across a pelt. A possible mechanism to explain the differences in THg concentrations observed in different anatomical and fur regions is varying levels of blood flow between these regions. Taylor & Minabe^24^ note that the structure of blood vessels in mammalian species is related to the mobility of the skin^24^. In general, skin with higher mobility due to greater functional demands had larger and longer blood vessels. Larger cutaneous blood vessels have the capacity to carry more blood and thus transport more Hg compounds at higher rates to the capillary beds that irrigate the hair follicles in these regions. As a result, skin regions with higher mobility may also excrete Hg compounds into fur from these regions at higher rates. Further, some of the interindividual variation could be explained by biological factors such as fur growth rate, fur growth period, seasonal molting and seasonal diet changes, which may contribute to the differences observed between the two fur types^25,26^. Additional information on the age and sex of the river otters in this study may have also been useful. However, there is no consensus in the literature as to whether age or sex affects the fur THg concentrations in river otters^16,27^.

The fraction of MeHg in THg was 95.7 ± 3.4% in topcoat fur. The ratio calculated in this study provided much higher precision than what has been reported MeHg in THg (78.6 ± 25.9%)^28^. The greater variability could have resulted from a mixture of topcoat and undercoat fur as no distinction was made. Compared to humans, where hair mean MeHg in THg ratios range from 79–94%, the MeHg in THg ratio in otter fur is within the high end of this range^29^. This indicates that the majority of Hg compounds in fur consist of MeHg and supports previous research that fur is an important excretory pathway for MeHg^10^. Given that MeHg and THg concentrations are also correlated in the brain, kidney and liver, this suggests that fur THg measurements can be used to estimate organ MeHg concentrations^17,28^.

In all individual and composite pelts, topcoat fur contained fewer significant hotspots and coldspots than the undercoat fur. In general, the head region typically had higher THg concentrations than tail. The occurrence of significant THg hotspots in the head and upper body region is consistent with the high THg measurement in fur sampled from the base of the skull in Wilkie *et al*.^17^. The existence of significant THg hotspots and coldspots in undercoat fur provides further evidence that undercoat fur concentrations are more variable than topcoat concentrations and suggests that heterogeneous regions may occur regularly in these locations on an average pelt. The average percent error for predicted organ THg was lower in the topcoat than the undercoat. Further, the cluster analysis of the average percent error demonstrates that errors are lowest around the head region of the topcoat and the tail region of the undercoat. This indicates that concentrations found in the head of the topcoat and the tail of the undercoat are similar and result in similar predictive accuracy. Therefore, using fur THg to predict internal THg via the conversion factors, are most accurate when samples are taken from the forebody region. Previous samples taken from other regions may be subject to increased rates of error, especially samples taken from the undercoat of the forebody.

Based on our results, we suggest the development of a standardized fur sampling protocol for biomonitoring THg in river otter. It is important this protocol balances the need for accurate biomonitoring with the economic considerations for trappers. Thus, limbs are optimal locations for sampling. The composite pelt and cluster analyses help to identify the optimal biological sample region. Topcoat is better suited for sampling as the THg concentrations are less variable than the undercoat. Further, samples should be taken from the head/forelimb region of the pelt to be most reflective of internal THg concentrations when using the conversion factor outlined in Eccles *et al*.^9^. Implications of estimating internal THg concentrations with fur sampled from other regions include the over or underestimation of the internal THg concentrations. These results also support the use of other non-invasive methods of fur sampling including the use of hair snares^30^. These samples are typically comprised of top coat from the head region. Thus, these samples would provide good estimates of internal THg concentrations and could be use in biomonitoring programs.

These results support the use of THg in the fur of river otters for biomonitoring programs to generate precise estimates of internal THg exposure based on fur THg concentrations when measured in topcoat fur in the fore body region. Simple sample collection protocols combined with the long-term stability of Hg in the fur matrix make this optimal for community-based monitoring programs to monitor Hg pollution in freshwater ecosystems. Future work includes assessing the distribution of THg of river otters with high mercury exposure to see if higher body burdens alter the distribution of THg across a pelt and assessing possible effects of age and sex on the distribution of THg across a pelt. Additionally, the characterization of THg distributions in other furbearing piscivorous mammals will be valuable for validating the use of fur biomonitoring programs in other sentinel species such as mink.

## Methods

The otter pelts and caresses were provided by Northern Alberta commercial trappers recruited through the Alberta Trappers Association. All animals were trapped under permit for the commercial fur trade following the Alberta Code for Responsible Trapping and the Agreement on International Humane Trapping Standards (AIHTS). Because the river otter pelts were collected under permit for the commercial fur trade following all appropriate ethics standards and were not as such considered “experimental animals”, Environment and Climate Change Canada’s Animal Care Committee (ACC) did not require any additional animal ethics or experimentation approvals.

### Pelt Processing

Dried whole otter pelts and frozen carcasses were obtained from a trapline located in northern Alberta, Canada. The pelts were flattened for sampling by cutting along the *ventral sagittal axis* (anus to middle of lower jaw). The dimensions of each pelt (*median sagittal axis*: length of nose to anus; *transverse axis*: midpoint between right fore/hind limb to midpoint between left fore/hind limb) were used to establish the proportions of the rectangular grid for each pelt, with the origin located at the intersection of the median sagittal and traverse axes. To account for the size difference between the pelts, the grid was scaled and then marked out using lab tape. Pictures of the gridded pelts can be seen in Supplementary Figs S1–S4. Topcoat and undercoat fur samples (50–150 mg) were taken from the middle of each rectangle. Both topcoat (technically referred to as guard hair) have the same hair follicle composition, comprised of an outer cuticle, a cortex, and a medulla (central core). However, the topcoat hairs are longer and coarser than undercoat^31^. The pictures of the difference between these two hair types can be seen in Supplementary Figs S5–S6.

### Mercury analysis

Approximately 10 mg of topcoat and undercoat were subsampled and total mercury was measured using a direct thermal decomposition Hg analyzer (Mercury Analyzer 3000; Nippon Instruments North America, Texas, USA). Quality assurance/quality control (QA/QC) methods included blanks samples, standard reference material (DORM-4 and IAEA-085), and 10% of all samples were duplicated. A stratified random subset based on THg concentration tertiles and anatomical region was used to select samples for MeHg analysis.

MeHg was measured using a method adapted from Cai (1997) and Laffont^32,33^. Each topcoat fur sample was first digested in 7.0 mL of trace metal grade 3.0 M HNO_3_ prepared with 18MΩ deionized water (DIW) for 16 hours at 55 °C in a clean glass vials with Teflon caps. Organic mercury species dissolved in the fur digestate solutions were extracted into 5 mL *Optima* grade dichloromethane (DCM), shaken for 24 hours on an orbital shaker at 330 rpm, and centrifuged at 3000 rpm for 10 mins. A known quantity of the DCM layer was transferred to a clean scintillation vial with 2.5 mL of 0.01M L-cysteine (Aldrich Chemical Company, Inc.) in DIW. This was shaken at 330 rpm for 45 mins, mixed for 20s on a Fischer Scientific *Vortex Mixer*, and centrifuged at 3000 rpm for 10 mins. Finally, 100 μL of the aqueous layer was spread on a thin layer of Additive B (Nippon Instruments Corporation #282–62665) and analyzed with the Mercury Analyzer 3000.

Organ tissues were dissected, mechanically homogenized (Heidolph Silent Crusher homogenizer; Sigma-Aldrich), and then freeze-dried for 48 h (FreeZone Freeze Dry System; Labconco). Approximately 50 mg of ground tissue were subsampled and analyzed using Mercury Analyzer 3000. QA/QC methods included blanks samples, standard reference material (DORM-4 and DOLT-5), and 10% of all samples were duplicated.

### Statistical analysis

All statistical analyses were performed in R 3.4.3^34^ using the packages car^35^, gstat^36^, lmtest^37^, sp^38^, and spdep^38^. A spatially, two- tailed paired and unpaired Student’s t-tests (equal variance) and Welch’s t-test (unequal variance) were used to evaluate differences in THg in the topcoat and undercoat and to evaluate differences in the THg in the right and left hindlimbs. F-tests for equality of variances were used to quantify the relative variability of THg concentrations between topcoat and undercoat. A one-way Analysis of Variance (ANOVA) was used to determine if there are regional differences in THg between anatomical region and fur region. The location of the anatomical region on each pelt can be seen in Supplementary Fig. S7 and the location of the fur regions on each pelt can be seen in Supplementary Fig. S8. A linear regression model was used to assess the relationship between the THg in the topcoat and undercoat.

Spatial analyses include Getis and Ord’s local *G*_i_* cluster analysis to identify regions with statistically significant high or low fur THg concentration^39^. A semi-variogram was used to determine the range autocorrelation, as known as a neighborhood. The range of spatial influence is quantified using the range of the semi-variogram. The calculated range distance was then used as the neighborhood parameter in the Getis and Ord’s Gi* analysis.

A linear regression was used to determine the relationship between MeHg and THg concentrations in topcoat fur. The model intercept was set to zero. Samples with MeHg in THg ratios greater than 110% were removed from the regression analysis to avoid bias due to analytical error^40^. Where necessary, models were tested for linearity (resettest: lmtest), homoscedasticity of residuals (ncvTest: car), residual normality (shapiro.wilks: lmtest) and no serial autocorrelation among residuals (dwtest: lmtest). All models presented met parametric test assumptions.

### Pelt Average

The individual pelts topcoat and undercoat were then normalized between zero and one (Eq. 1). Points with the same coordinates were averaged to produce an average normalized THg value for both the topcoat and undercoat. Using the same methods outlined in the statistical analyzes, the composite was analyzed for regional differences and hotspots.

1 $${{c}}_{{i}{normalized}}=\frac{{{c}}_{{i}}-{{c}}_{{\min }}}{{{c}}_{{\max }}-{{c}}_{{\min }}}$$

### Optimal Sample Location

Using the conversion between fur THg and organ THg developed in Eccles *et al*.^9^, the brain, kidney, liver, and muscle concentrations were estimated based on the THg concentration at every sample point in the topcoat and undercoat. The percent error was calculated for each organ at each sample point for topcoat and undercoat (Eq. 2).

2 $$ \% {Erro}{{r}}_{{i}}=({abs}(\frac{{\hat{y}}_{i}-{{y}}_{{i}}}{{{y}}_{{i}}}))\ast 100$$

Then a weighted average error for all organs in the topcoat and undercoat was calculated, where the brain had a weighting twice that of other organs due to its importance in mercury toxicity. Using the same methods outlined in the statistical analyzes, the composite error rates was analyzed for hotspots to identify the region of the pelt with the lowest error.

## Supplementary information

S1: Results and Methods

## Data Availability

Data is available upon request from the authors. R code used in these analyses can be found at https://github.com/kristineccles/pelt_hg.git.
