# Supplementary material for: Distribution of organic and inorganic mercury across the pelts of Canadian river otter (Lontra canadensis)
Source: Sci Rep. 2019 Mar 1;9:3237. doi: 10.1038/s41598-019-39893-w (PMC6397270; doi:10.1038/s41598-019-39893-w)
Supplement: Supplementary file 1 — S1: Results and Methods [file 41598_2019_39893_MOESM1_ESM.docx]

**Title:** Distribution of organic and inorganic mercury across the pelts of Canadian river otter (*Lontra canadensis)*

**Authors:** Kristin M Eccles^1^, Eric S. Littlewood^1^, Philippe J Thomas^1,2^, Hing Man Chan^1*^

**Affiliations:**

^1^Department of Biology, University of Ottawa, 180, Gendron Hall, 30 Marie Curie, Ottawa, ON, K1N 6N5, Canada

^2^ Science and Technology Branch, Environment and Climate Change Canada, National Wildlife Research Center, 1125 Colonel By Drive, Raven Road, Ottawa, ON K1A 0H3, Canada

**Email Addresses:** [kristin.eccles@uottawa.ca](mailto:kristin.eccles@uottawa.ca), elittw@uottawa.ca [philippe.thomas@canada.ca](mailto:philippe.thomas@canada.ca), [laurie.chan@uottawa.ca](mailto:laurie.chan@uottawa.ca)

**Corresponding author:** Laurie H.M. CHAN

180, Gendron Hall, 30 Marie Curie, Ottawa, ON, K1N 6N5

Tel: (613) 562-5800 (7116) Fax: (613) 562-5385

Email: [laurie.chan@uottawa.ca](mailto:laurie.chan@uottawa.ca)

**S1: Results and Methods**

**Table S1.** Equality of variances F-test results for topcoat (TC) and undercoat (UC) samples for individual pelts.

| **Pelt #** | **Ratio of the variances**  **(TC/UC)** | **F-value**^1^ | **p-value** |
| --- | --- | --- | --- |
| Pelt 1 | 0.1005 | 0.1005 (88,88) | < 0.001 |
| Pelt 2 | 0.0084 | 0.0084 (97,97) | < 0.001 |
| Pelt 3 | 0.0617 | 0.0617 (95,95) | < 0.001 |
| Pelt 4 | 0.0331 | 0.0331 (94,94) | < 0.001 |
| ^1^Bracketed values are the degrees of freedom of the numerator and the denominator, respectively. | | | |

**Table S2.** Student’s t-test results for paired topcoat (TC) and undercoat (UC) samples with unequal variances for individual pelts.

| **Pelt #** | **Mean of the differences (TC - UC) (ppm)** | **t statistic**^1^ | **p-value** |
| --- | --- | --- | --- |
| Pelt 1 | -0.6 | -9.56 (88) | < 0.001 |
| Pelt 2 | -4.8 | -22.3 (97) | < 0.001 |
| Pelt 3 | -1.4 | -9.82 (95) | < 0.001 |
| Pelt 4 | -0.4 | -4.83 (94) | < 0.001 |
| ^1^Bracketed values are degrees of freedom | | | |


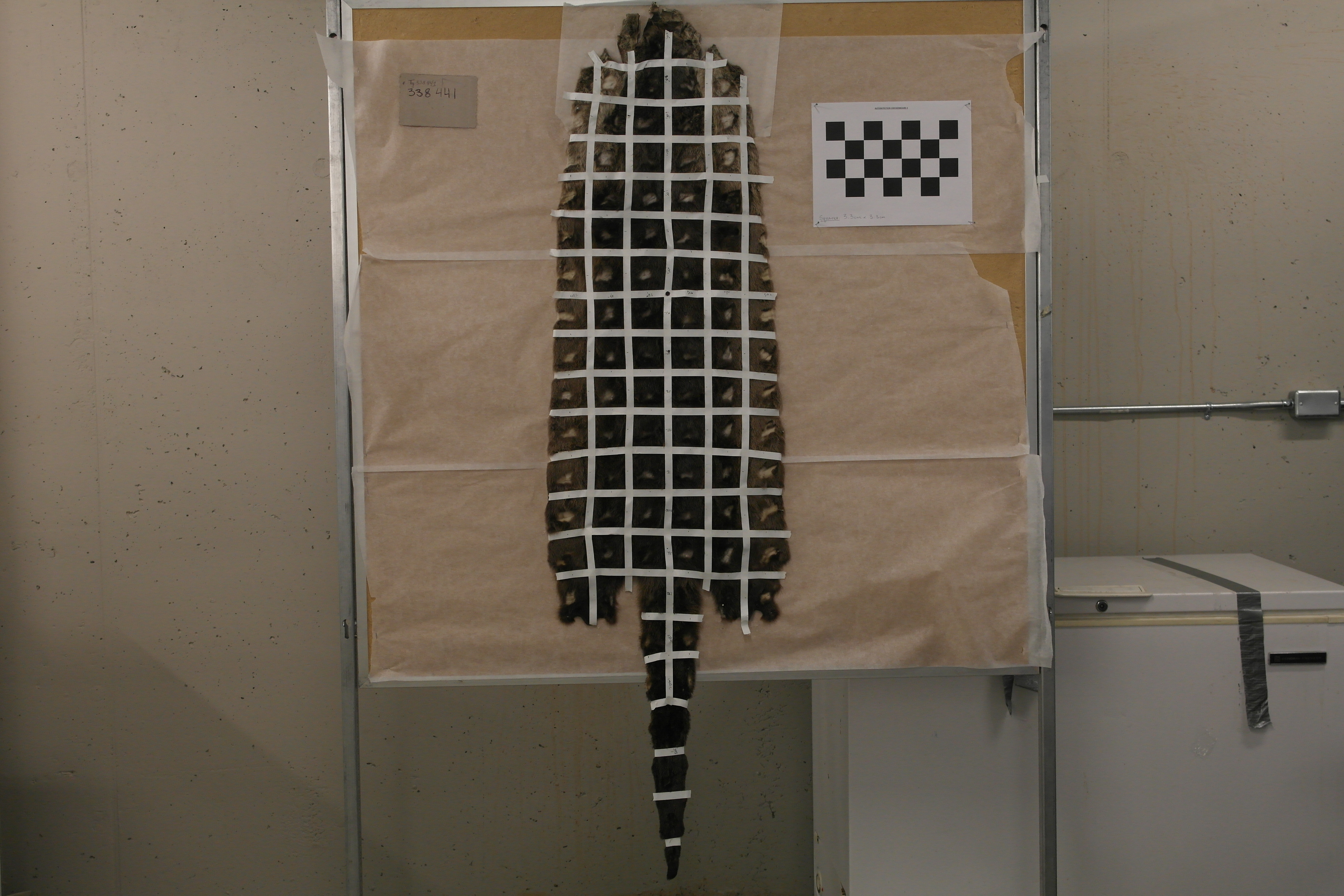


Figure S1. Photo of gridding for pelt 1.


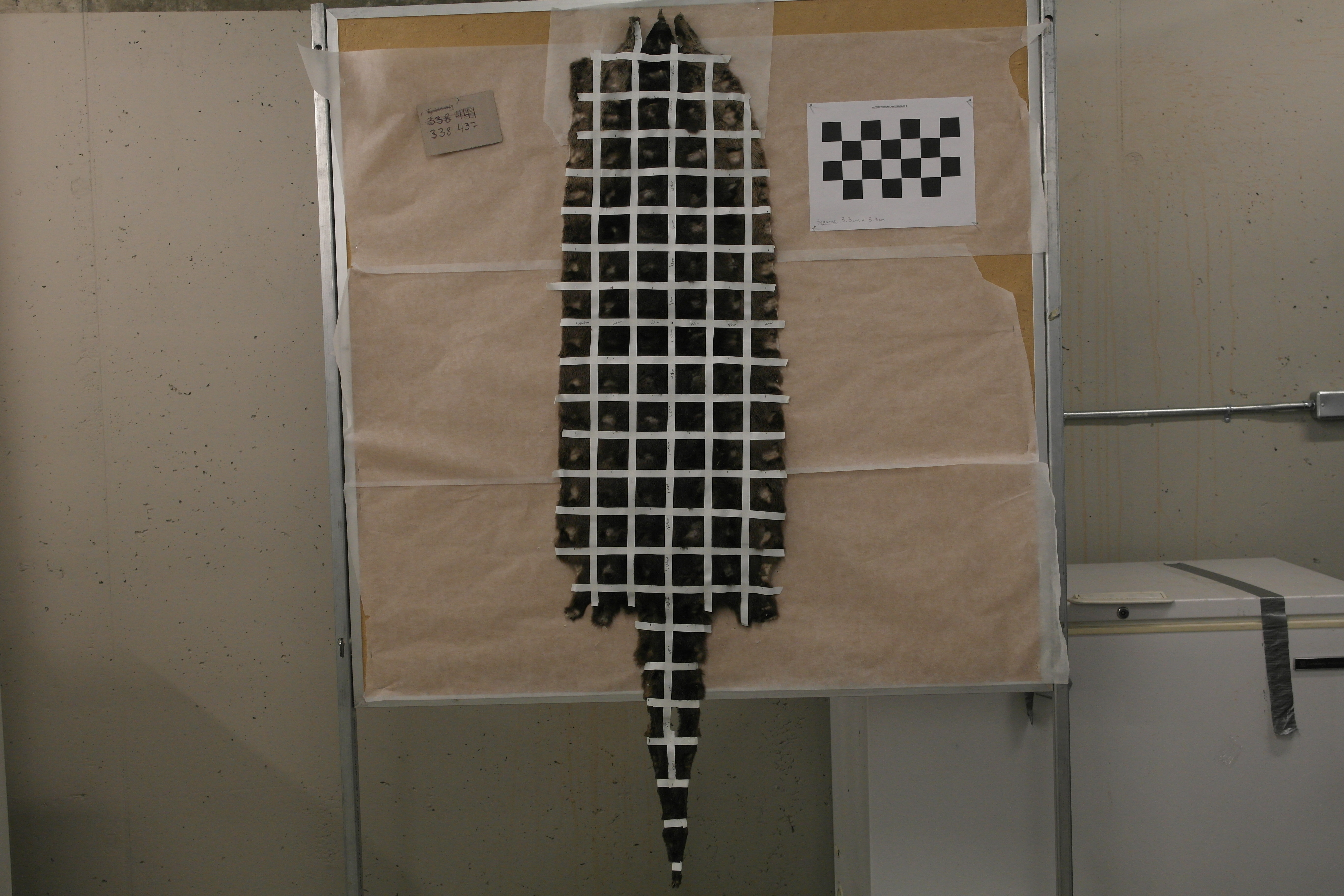


Figure S2. Photo of gridding for pelt 2.


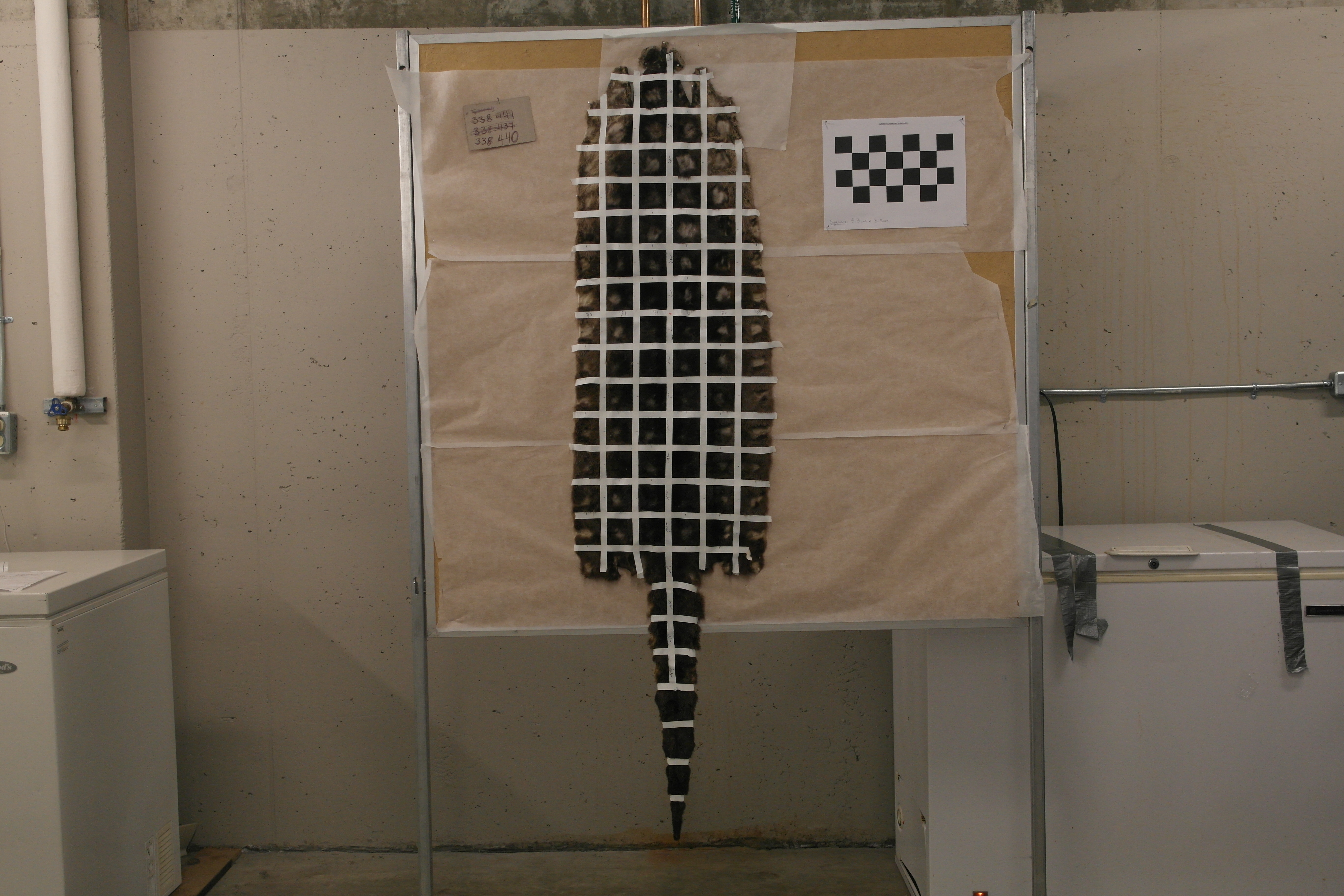


Figure S3. Photo of gridding for pelt *3*.


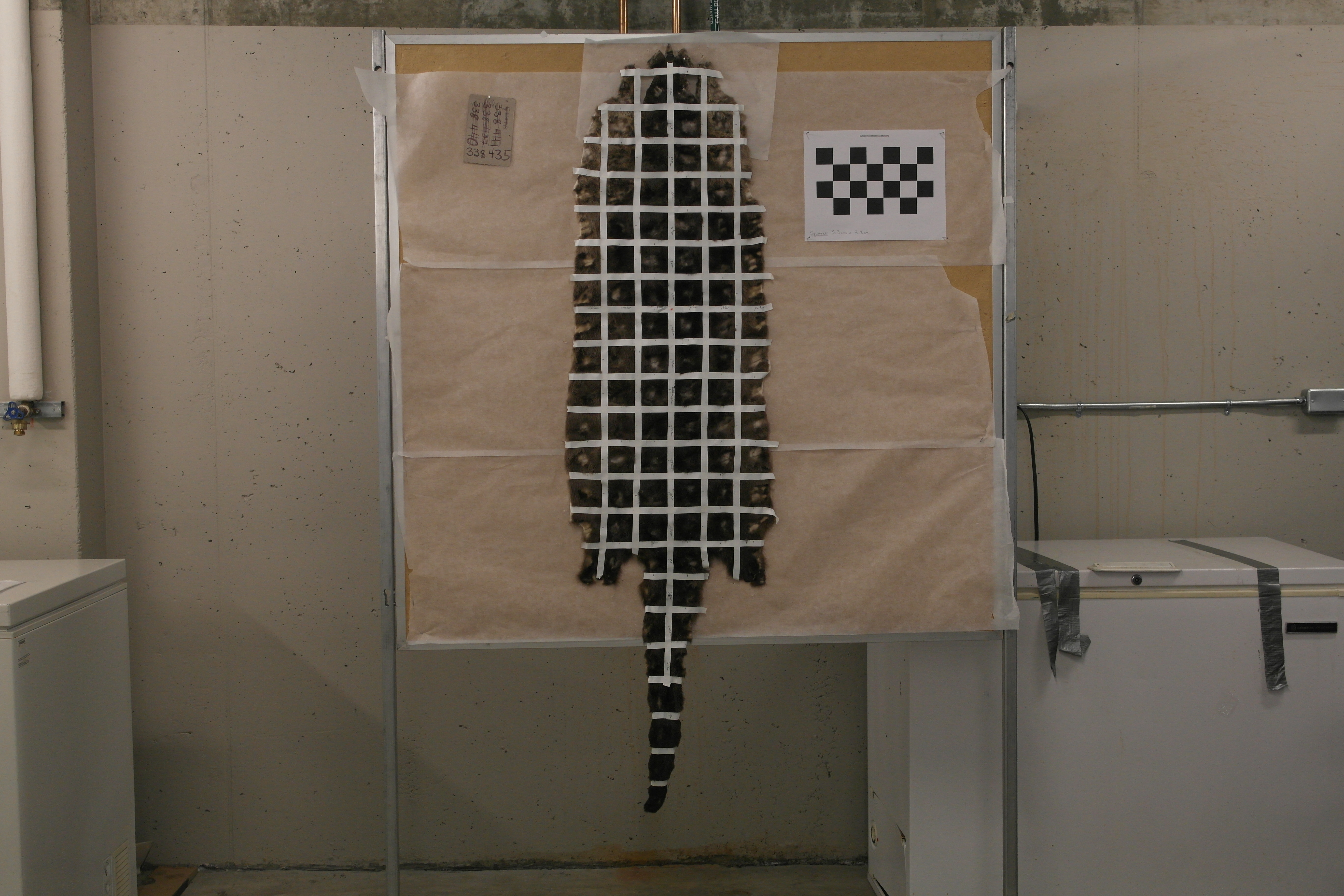


Figure S4. Photo of gridding for pelt 4.


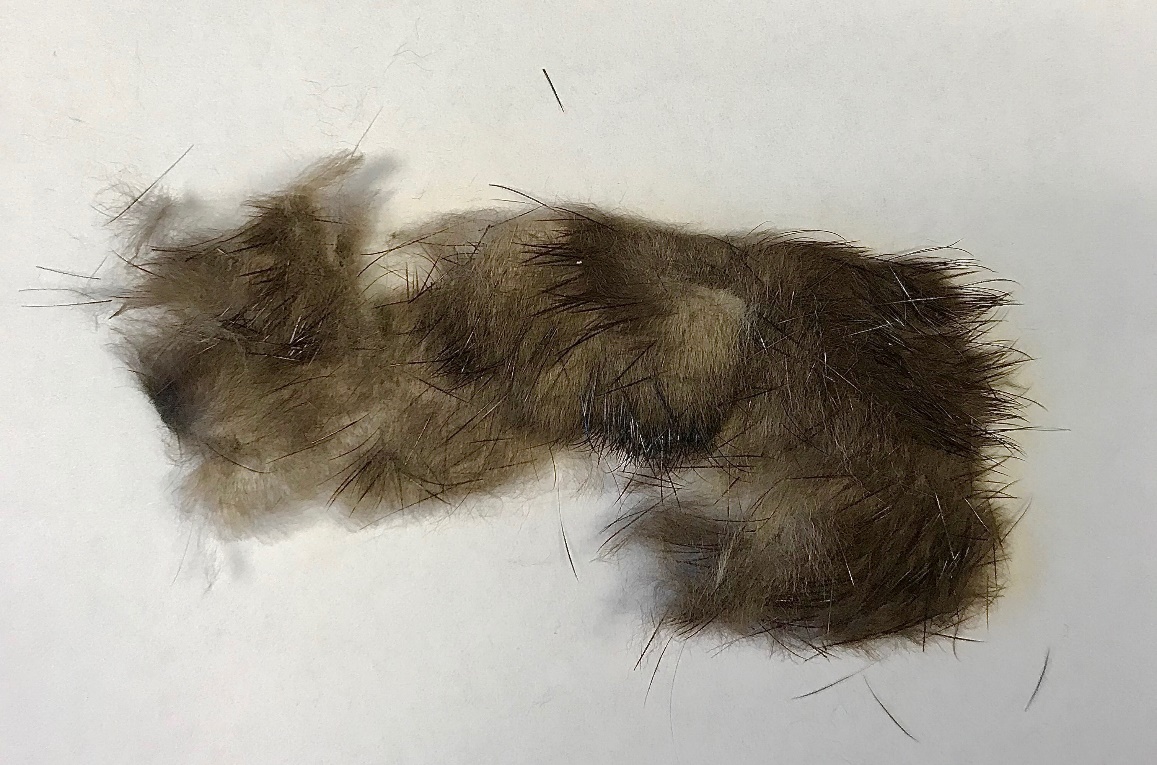


Figure S5. Photo of fur sample after being removed from the pelt with topcoat (dark coarse hair) and undercoat (lighter thin hair).


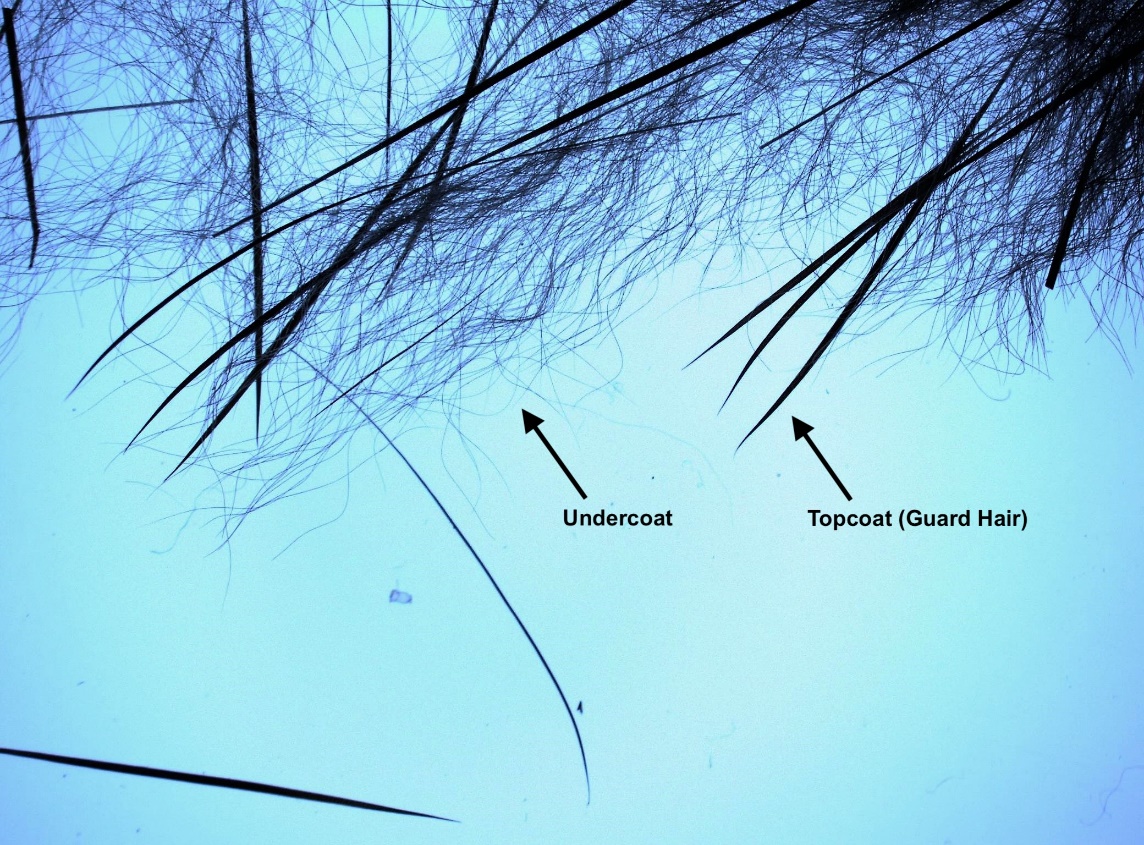


Figure 6. Photo of fur sample under a dissecting microscope to show the difference between topcoat and undercoat fur; the topcoat is coarser and longer and undercoat is shorter and thinner.


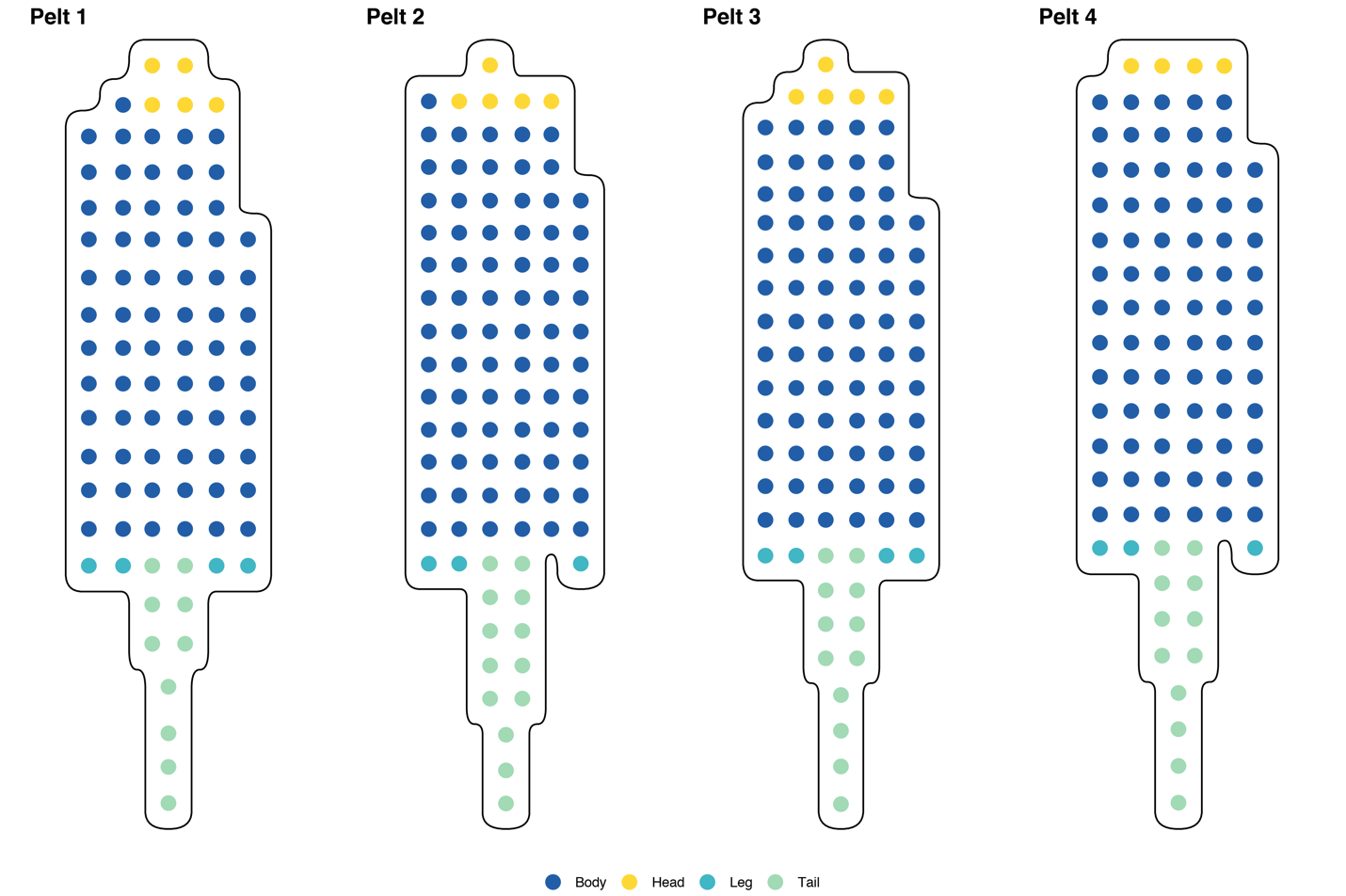


Figure S7. A plot illustrating the different anatomical regions used for analysis on the pelt each.


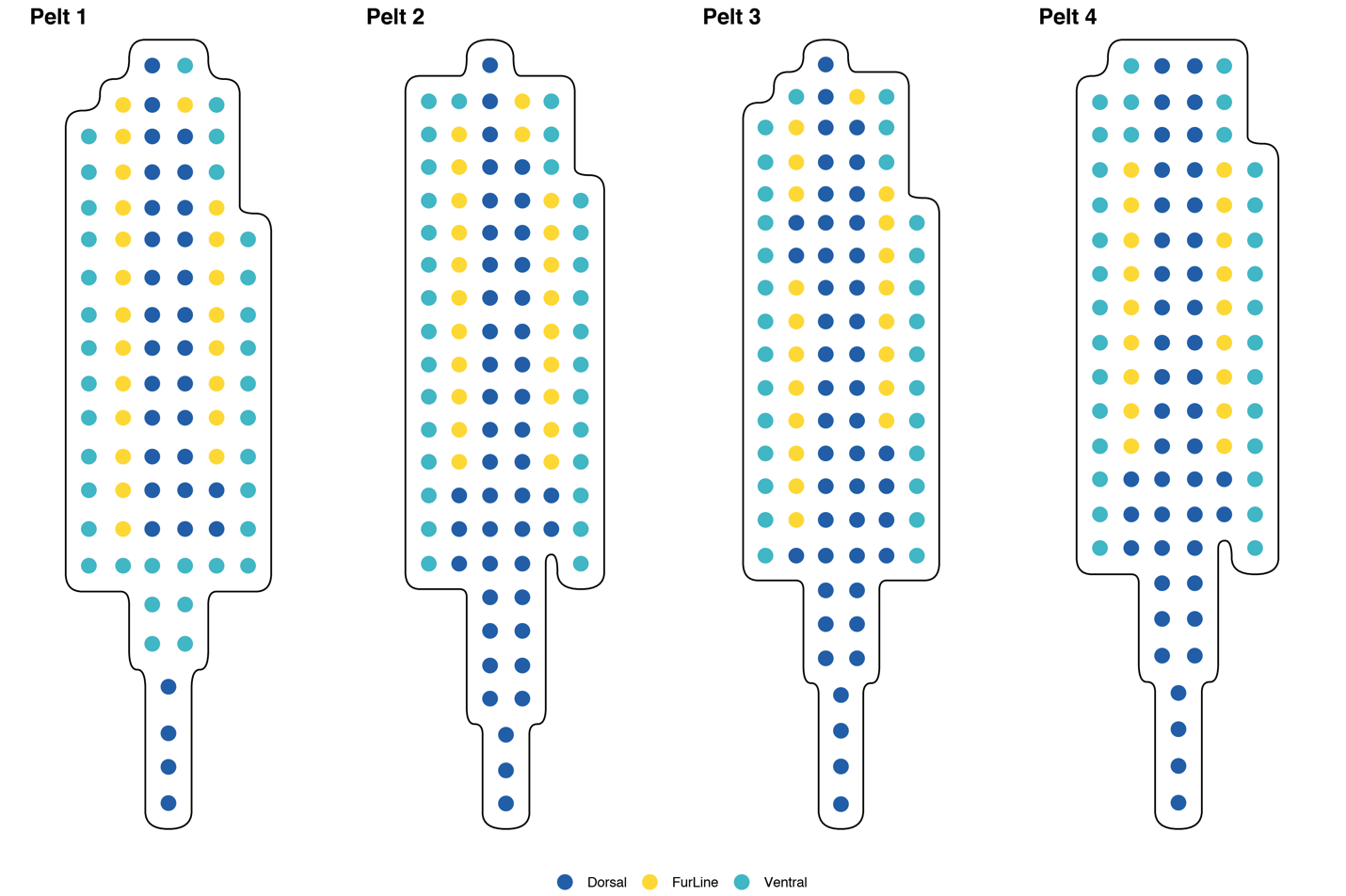


Figure S8. A plot illustrating the different fur regions used for analysis on the pelt each.
